# Overcoming barriers: mechanisms and strategies of nanoparticles in overcoming the blood-brain barrier and drug resistance in glioblastomas

**DOI:** 10.1186/s43046-026-00362-x

**Published:** 2026-05-18

**Authors:** Qiusi Tian, Yanbin Liang, Tao Huang, Feilong Chen, Qun Zhang

**Affiliations:** 1 Department of Neurosurgery, 3201 Hospital, ShaanXi, China; 2 Chengdu Sport University, Chengdu, China; 3 Shaanxi University of Technology, Hanzhong, China

**Keywords:** Glioma, Blood-brain barrier, Drug resistance, Nanoparticles, Drug delivery, Tumor microenvironment, Photothermal therapy, Ferroptosis

## Abstract

Glioma, particularly glioblastoma (GBM), represent a major challenge in neuro-oncology due to their highly aggressive nature and poor therapeutic outcomes. The presence of the blood-brain barrier (BBB) severely restricts the efficient delivery of therapeutic agents to tumor sites, while the frequent emergence of drug resistance further compromises treatment efficacy.

In recent years, nanoparticles have emerged as promising drug delivery platforms, demonstrating considerable potential in overcoming the BBB, enhancing targeting specificity, and reversing drug resistance, owing to their favorable biocompatibility and tunable physicochemical properties. This review provides a comprehensive overview of recent advances in the application of nanoparticles for glioma therapy, with a particular focus on strategies for BBB penetration and resistance reversal.

Specifically, we summarize the design principles and functional modifications of nanoparticles, as well as their diverse therapeutic mechanisms, including drug delivery, photothermal and photodynamic therapy, ferroptosis induction, and modulation of the tumor microenvironment. In addition, the mechanisms underlying nanoparticle-mediated targeting and controlled drug release are discussed in detail.

Importantly, current evidence suggests that nanoparticle-based platforms offer a versatile and effective approach for overcoming key biological barriers and therapeutic resistance in glioma, although their clinical translation remains constrained by challenges related to safety, scalability, and targeting heterogeneity. Future research should focus on the development of multifunctional and personalized nanomedicine strategies, integration with multimodal therapies, and rigorous evaluation of long-term safety and clinical efficacy, in order to facilitate the successful translation of these technologies into clinical practice.

## Introduction

Glioma, particularly glioblastoma multiforme (GBM), is the most common and aggressive brain tumor, posing significant challenges in clinical treatment and resulting in a poor prognosis, with a median survival of less than 15 months. Conventional therapeutic approaches, including surgical resection, radiotherapy, and chemotherapy, remain the standard of care; however, their efficacy is limited by high recurrence rates and therapeutic resistance [1, 2].

One of the primary challenges in glioma treatment is the presence of the blood-brain barrier (BBB), a highly selective physiological barrier formed by tightly connected brain microvascular endothelial cells. The BBB strictly regulates the transport of substances between the bloodstream and brain tissue, thereby significantly restricting the delivery of most chemotherapeutic agents and biological therapeutics to tumor sites [3, 4]. In addition, the tumor microenvironment, glioma stem cells, and drug resistance mechanisms further compromise therapeutic efficacy [5, 6]. Collectively, these factors contribute to the poor sensitivity of glioma to conventional therapies and promote tumor recurrence and progression.

Drug resistance in glioma is a complex and multifactorial process involving multiple molecular pathways. For instance, the DNA repair enzyme O6-methylguanine-DNA methyltransferase (MGMT) can reverse DNA damage induced by alkylating agents, thereby reducing tumor cell sensitivity to chemotherapy [7]. Furthermore, the upregulation of multidrug resistance-associated proteins, the self-renewal capacity of tumor stem cells, and the accumulation of immunosuppressive cells within the tumor microenvironment all contribute to treatment resistance [8, 9]. Immune cells such as tumor-associated macrophages (TAMs) and myeloid-derived suppressor cells (MDSCs) further facilitate immune evasion and resistance to therapy [10, 11].

Recent advances in nanotechnology have provided new opportunities for glioma treatment. Nanoparticles, characterized by tunable physicochemical properties, enable targeted delivery across the BBB, enhance drug stability, and improve therapeutic efficacy [12, 13]. Functionalized lipid, polymer, and metal nanoparticles have shown effective brain targeting and anti-tumor activity in drug delivery studies [14, 15]. Moreover, nanocarriers can simultaneously deliver multiple therapeutic agents, including chemotherapeutic drugs, small interfering RNA (siRNA), and immunomodulators, thereby enabling combination therapy and enhancing treatment outcomes [16, 17].

Surface modification strategies further improve the targeting efficiency of nanoparticles. By functionalizing nanoparticles with tumor-targeting ligands, such as transferrin receptor ligands, tumor-specific antibodies, and peptides, receptor-mediated transport across the BBB can be achieved, leading to enhanced accumulation in tumor tissues [18, 19]. In addition, biomimetic nanoparticles coated with tumor cell membranes exhibit prolonged circulation time, immune evasion capability, and homologous targeting properties, thereby improving delivery specificity and efficiency [20, 21].

Furthermore, nanotechnology enables the regulation of the tumor microenvironment through emerging therapeutic strategies, including immunotherapy, targeted stem cell therapy, and gene therapy, offering multiple avenues for combination treatment [22, 23]. Therefore, nanoparticle-based platforms provide versatile approaches for overcoming the BBB and drug resistance, which are the major obstacles in glioma therapy [3, 24].

In summary, the presence of the BBB and the development of drug resistance remain critical barriers to effective glioma treatment. Nanotechnology offers innovative solutions by enhancing drug delivery, improving targeting specificity, and modulating tumor biology. This review focuses on the mechanisms by which nanoparticles overcome the BBB and reverse drug resistance, aiming to provide insights into the rational design and future clinical translation of nanomedicine for glioma therapy.

## How nanoparticles overcome the blood-brain barrier: mechanisms and design strategies

### Structural and functional limits of the blood-brain barrier

The BBB is a selective barrier in the central nervous system (CNS), composed of endothelial cells, that separates the blood from brain tissue, maintains the stability of the brain microenvironment, and protects against harmful substances [25]. The structure of the BBB consists of tight junctions between endothelial cells, the basement membrane, and astrocyte end-feet, forming a highly restrictive barrier that limits molecular transport [26, 27]. The basement membrane provides structural support and influences endothelial cell function, while astrocyte end-feet surround blood vessels and regulate permeability and barrier integrity [28, 29]. Additionally, pericytes are also an important component of the BBB, playing a key role in maintaining vascular stability and barrier function [27].

The functional limitations of the BBB are mainly reflected in its highly selective transport capacity, which restricts the entry of most drugs and exogenous molecules while relying on transport proteins and metabolic enzymes for regulation [30, 31]. Among these, ATP-binding cassette transporters, like P-glycoprotein, prevent drug and toxin accumulation in the brain [32]. Furthermore, the glycocalyx on endothelial cells regulates vascular permeability and inflammatory responses; its disruption can increase BBB permeability, leading to brain edema and neuronal damage [33].

**Fig. 1:**
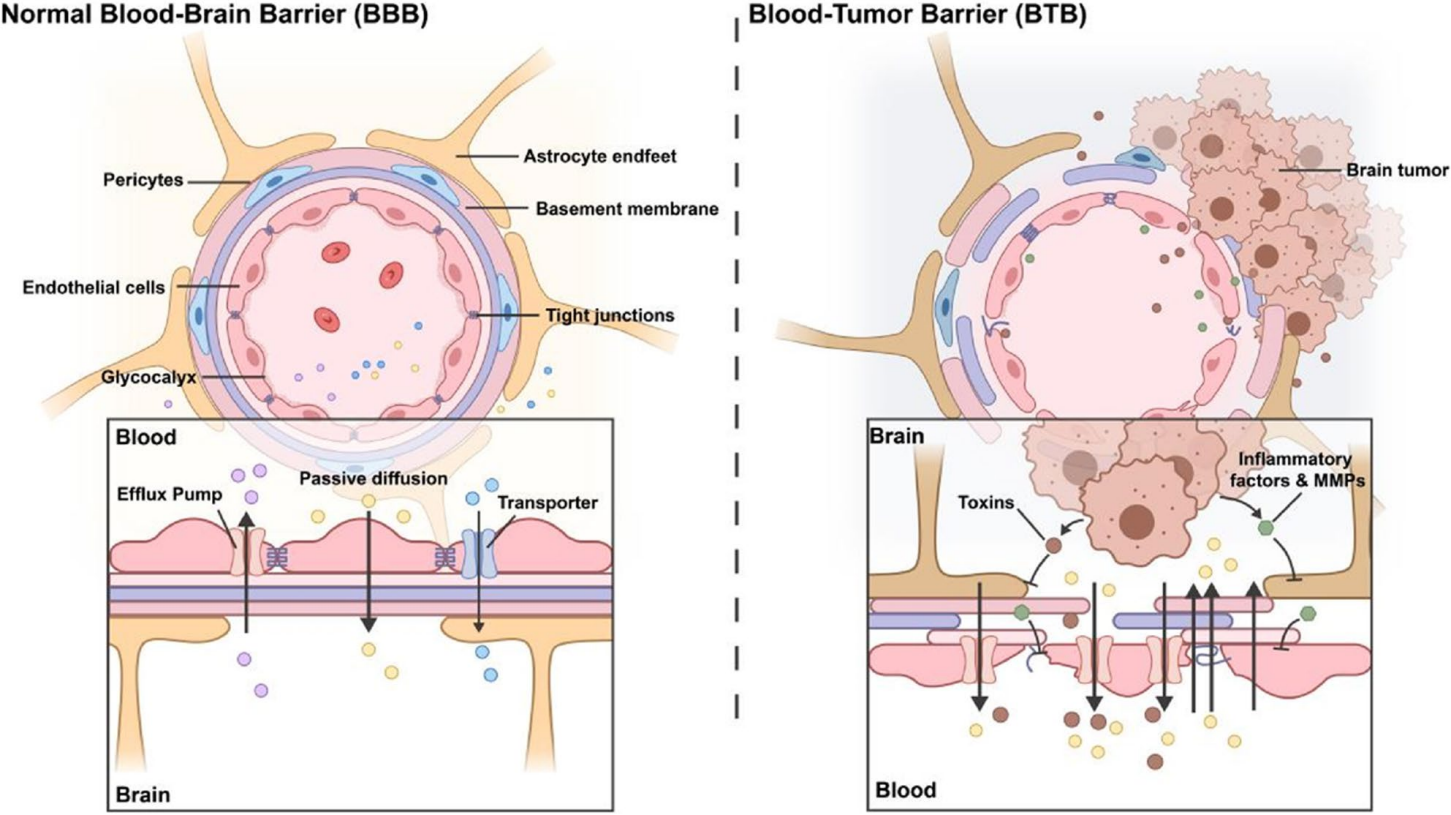
Schematic comparison of the structure and transport characteristics of the normal BBB and the blood -tumor barrier (BTB) in glioma. The normal BBB (left) exhibits an intact and highly selective structure that maintains brain homeostasis and restricts harmful substances. In contrast, the BTB (right) shows disrupted barrier integrity, increased permeability, and heterogeneous structural alterations, which, together with upregulated efflux transporters, limit effective drug delivery to tumor tissues

In the tumor microenvironment of brain tumors such as glioma, the structure and function of the BBB undergo dynamic changes [34]. On one hand, tumor-associated vasculature forms the blood -tumor barrier (BTB), which is more permeable than the normal BBB but still limits the entry of chemotherapeutic agents.On the other hand, inflammatory factors and matrix metalloproteinases (MMPs) can disrupt tight junctions, resulting in heterogeneous barrier breakdown and localized leakage [25, 35]. Additionally, the drug resistance mechanisms in brain tumors, such as the upregulation of P-gp, further limit drug accumulation in tumor tissues [32]. Therefore, the BBB and BTB together present significant obstacles to effective drug delivery in glioma treatment.The structural and functional differences between the normal BBB and BTB are illustrated in Fig. 1.

In summary, the BBB is composed of endothelial cells, the basement membrane, astrocyte end-feet, and pericytes, and exhibits highly selective permeability, which poses major challenges for drug delivery in glioma therapy. Understanding its structural characteristics and dynamic alterations in tumors is essential for developing effective strategies to overcome drug resistance [25–27, 33–35].

### Regulation of the physicochemical properties of nanoparticles

The physicochemical properties of nanoparticles play a critical role in determining their ability to cross the BBB and achieve effective drug delivery [36]. Key factors include particle size, surface charge, shape, and surface modification, all of which influence circulation time, cellular uptake, and biodistribution [37, 38].

Particle size is one of the most important parameters affecting BBB penetration. Generally, nanoparticles within an appropriate size range are more likely to cross the BBB, whereas excessively large particles are restricted, and very small particles are rapidly cleared from circulation [39, 40]. In addition, size also influences cellular internalization and accumulation in tumor tissues, thereby affecting therapeutic outcomes.

Surface charge is another important factor that affects nanoparticle interaction with the BBB. Positively charged nanoparticles can enhance interaction with negatively charged cell membranes, thereby facilitating cellular uptake; however, they may also lead to increased cytotoxicity and rapid clearance from the bloodstream [41, 42]. In contrast, neutral or slightly negatively charged nanoparticles tend to exhibit improved biocompatibility and longer circulation times, although their cellular uptake efficiency may be relatively lower.

The shape of nanoparticles also affects their biological behavior. Spherical nanoparticles are generally more readily internalized by cells, whereas rod-shaped or other anisotropic nanoparticles may exhibit prolonged circulation time and different cellular uptake pathways [43, 44]. These differences can influence their ability to cross the BBB and accumulate in tumor tissues.

Surface modification is an effective strategy to improve the BBB penetration and targeting capability of nanoparticles. For example, polyethylene glycol (PEG) modification can enhance nanoparticle stability and prolong circulation time by reducing protein adsorption and immune recognition [45]. In addition, the introduction of targeting ligands, such as peptides, antibodies, or receptor-binding molecules, can facilitate receptor-mediated transport across the BBB and improve tumor-specific accumulation [46, 47].

In summary, the physicochemical properties of nanoparticles, including size, charge, shape, and surface characteristics, significantly influence their transport across the BBB and therapeutic performance. Rational design and optimization of these properties are essential for enhancing delivery efficiency and achieving improved outcomes in glioma treatment [36–40, 48, 49].

### Active Targeting and Responsive Release Mechanisms

In glioma treatment, the active targeting and responsive release mechanisms of nanoparticles are essential strategies for achieving precise drug delivery and enhancing treatment efficacy. The glioma microenvironment exhibits variousseveral unique triggers, including aselevated highlevels of glutathione (GSH), acidic conditions (decreased pH), and specific enzymes, which provide perfectan conditionsideal basis for designing the design of “smart” nanoparticles. By leveraging these characteristics of the tumor microenvironment, nanoparticles can selectively release therapeutic agents, thereby improving therapeutic outcomes and while reducing minimizing sideadverse effects on normal tissues.

In response to the elevated glutathione concentration in the tumor microenvironment, nanoparticles can be engineered with disulfide bonds that degrade down in a reducing environments. Due to the elevated intracellular glutathione (GSH) levels in glioma cells, disulfide bonds can be cleaved under reductive conditions, thereby promoting controlled drug release, preventing premature leakage, and enhancing therapeutic efficacy at the target site [50]. Likewise, nanoparticles composed of polymer materials featuring thioether or disulfide bonds can enable rapid drug release in the reducing environment prevalent within tumor cells, thereby improving drug targeting efficiency and therapeutic outcomes.

Secondly, the tumor-specific acidic microenvironment serves as a responsive cue. Multifunctional nanoparticles featuring acid-sensitive Schiff base structures can decompose in acidic conditions, facilitating rapid drug release. For example, a nanoparticle drug delivery system derived from dopamine derivatives can achieve active targeting through surface modification with folic acid, while also allowing for pH-responsive release of the encapsulated drug, thereby significantly enhancing drug uptake and inhibition in tumor cells [51]. Furthermore, nanoparticles modified with pH-responsive materials, such as carboxymethyl chitosan, demonstrate increased drug release rates in the acidic tumor microenvironment, thereby improving the precision of targeted therapy [52].

Enzyme responsiveness serves as a critical mechanism for intelligent drug release. Enzymes prevalent in the tumor microenvironment, such as hyaluronidase and matrix metalloproteinases, can initiate drug release. For instance, drug-loaded hyaluronic acid-bilirubin nanoparticles can rupture and release their payload in the presence of elevated levels of reactive oxygen species (ROS) within tumors. The hyaluronic acid shell simultaneously facilitates active targeting of CD44 high-expressing tumor cells, thereby significantly enhancing therapeutic efficacy [53]. Additionally, nanoparticles responsive to ROS and glutathione have been engineered for the tumor microenvironment, enabling stimulus-triggered drug release and demonstrating potential for synergistic therapeutic effects in glioma [54].

In summary, the integration of active targeting and responsive release mechanisms makes the most of the unique triggers of the tumor microenvironment, as well as external physical stimuli. This approach combines multimodal imaging and therapy to develop efficient and intelligent nanoparticle drug delivery systems. These strategies not only surmount the challenges posed by the blood-brain barrier and tumor heterogeneity but also effectively tackle issues of drug resistance, thereby offering robust technical support and guidance for advancements in the precise treatment of glioma [50, 51, 53, 55–57].

**Fig. 2:**
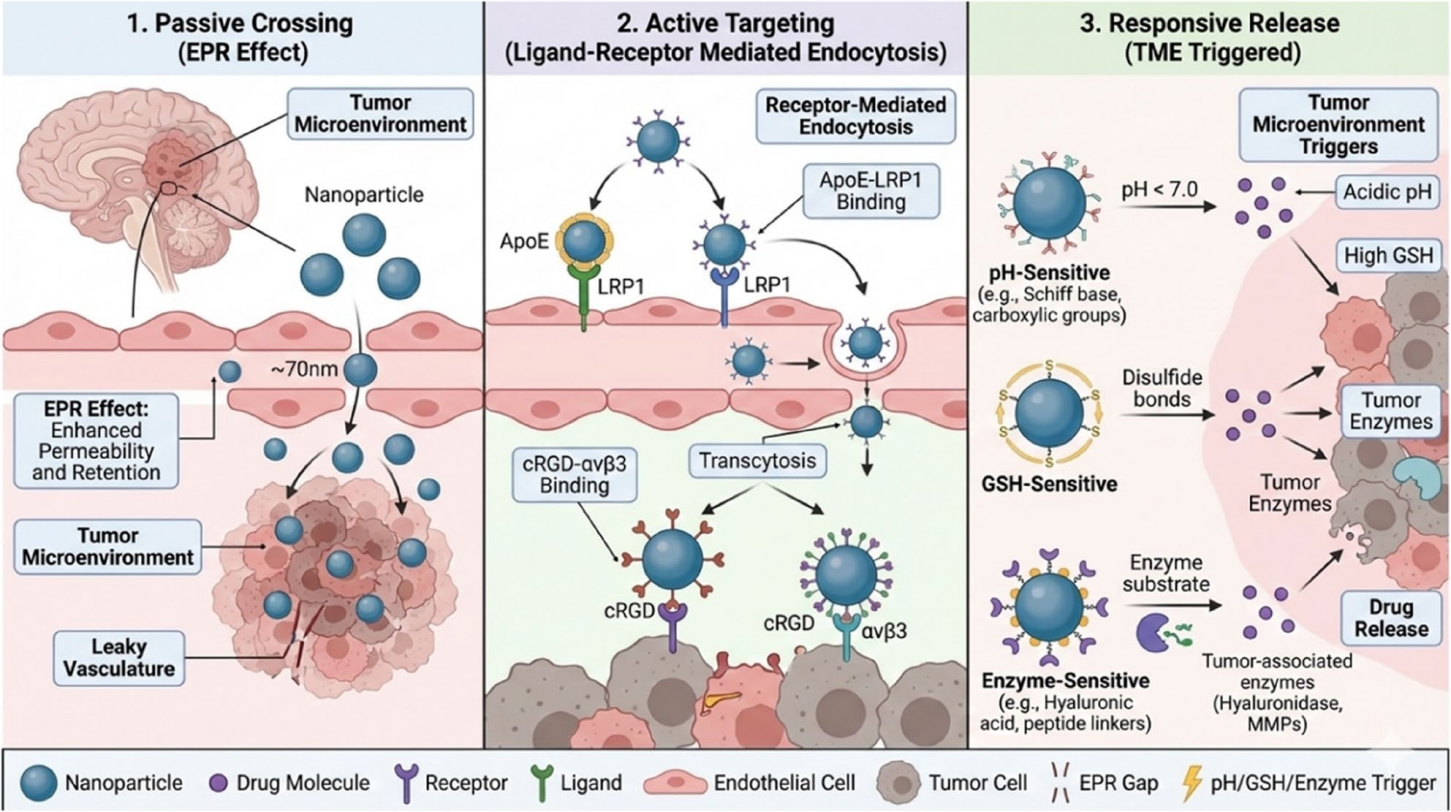
Schematic illustration of three nanoparticle-based drug delivery strategies for brain tumors: passive targeting via the enhanced permeability and retention (EPR) effect, active targeting through ligand -receptor interactions, and tumor microenvironment-responsive drug release

Figure 2 illustrates three main delivery strategies for nanodrugs in brain tumors.

### Typical nanomaterials and their application examples

Typical nanomaterials encompass metal nanoparticles, including gold and selenium nanoparticles, mesoporous silica nanoparticles, polymer nanoparticles, and lipid nanoparticles. These materials not only traverse the BBB efficiently but also target glioma cells for drug delivery, thereby enhancing drug bioavailability and minimizing systemic toxicity.

Gold nanoparticles (AuNPs), have attracted significant attention due to their biocompatibility and ease of functionalization. Functionalized AuNPs can deliver biomolecules, such as antibodies and siRNA, across the blood–brain barrier (BBB), enabling targeted glioma therapy. Notably, AuNPs enhance radiosensitivity by suppressing CCL2 expression via the TRAF6/NF-κB pathway [58].TeSe nanoparticles represent another promising metal nanoplatform, capable of inducing ferroptosis and calcium overload in glioma cells. Transferrin modification facilitates their BBB penetration, highlighting their therapeutic potential [59].

Mesoporous silica nanoparticles (MSNs) exhibit highly controllable pore structures and large specific surface areas, rendering makes them ideal for the loading of various drug molecules intended for controlled release and targeted delivery. Through the surface modification with specific ligands, MSNs can effectively recognize glioma cells, which enhances their accumulation and penetration at tumor sites, thus overcoming the limitations posed by the BBB.

Polymer nanoparticles, particularly those composed of PLGA (poly(lactic-co-glycolic acid)), are frequently employed for drug delivery in glioma treatment due to their favorable degradation properties and high drug-loading capacity. Studies demonstrate that PLGA nanoparticles can effectively transport the non-steroidal anti-inflammatory drug indomethacin, markedly increasing its inhibitory effect on glioma cells while facilitating efficient drug release via cellular phagocytosis [60].

Lipid nanoparticles (LNPs), which serve as nanocarriers that emulate cell membrane structures, demonstrate a remarkable capacity to traverse the BBB while exhibiting low immunogenicity. In recent years, LNPs have gained prominence for the delivery of nucleic acid therapeutics, such as small interfering RNA (siRNA). For instance, biomimetic nanomaterials derived from extracellular vesicles of glioma cells have facilitated efficient transport and targeted delivery of STAT3-siRNA, resulting in a significant reduction in glioma cell proliferation [61]. Moreover, by incorporating tumor-targeting ligands, lipid nanoparticles can effectively identify and treat residual glioma post-surgery, thereby delaying tumor recurrence [21].

In summary, conventional nanomaterials facilitate the traversal of the BBB and mitigate drug resistance in glioma due to their tunable structures and biocompatibility, thereby promoting advancements in glioma therapy. Future developments in targeted nanodelivery systems are expected to enhance both the efficacy and safety of glioma treatments.

## Mechanisms of nanoparticles for reversing drug resistance in glioma

### Molecular mechanisms of glioma drug resistance

Glioma drug resistance remains a major clinical challenge driven by complex molecular mechanisms. The MGMT (O6-methylguanine-DNA methyltransferase) gene plays a central role by repairing O6-methylguanine lesions induced by temozolomide (TMZ), thereby reducing its therapeutic efficacy. Approximately half of glioblastomas and two-thirds of grade II-III gliomas lack MGMT expression and are initially sensitive to TMZ, but often acquire resistance due to mismatch repair (MMR) deficiency [9]. In addition, MGMT-derived peptides (e.g., MGMT-02 and MGMT-04) are closely associated with TMZ resistance, exhibiting decreased apoptosis and enhanced resistance in vitro and in vivo, suggesting their potential as precise therapeutic targets [62].

Other components of DNA repair also contribute to therapeutic resistance. RECQL4 is overexpressed in glioma and promotes proliferation and resistance, whereas its depletion increases TMZ sensitivity and impairs tumor stem cell self-renewal, highlighting its role in replication stress and DNA repair [63]. PARP1, regulated by EZH2, represents another key factor; EZH2 overexpression upregulates PARP1 and enhances TMZ resistance, while dual inhibition of EZH2 and PARP1 markedly increases TMZ cytotoxicity [64]. In addition, RNA modifications, including m6A and m5C, facilitate adaptive resistance by modulating post-transcriptional regulation and gene expression programs [65].

Tumor stem cell characteristics (GSCs) significantly contribute to glioma resistance. Stem-like glioma cells demonstrate robust self-renewal and multi-directional differentiation capabilities, along with heightened tolerance to chemotherapy. Research indicates that CXCR4 enhances GSC proliferation and resistance by activating the downstream KLF5/BCL2L12 signaling pathway. The combination of CXCR4 inhibitors with TMZ effectively reverses resistance and improves anti-tumor effects [66]. Additionally, NRP1 increases the stemness of glioma cells and their resistance to TMZ by activating the Hippo pathway effector YAP [67]. Furthermore, KIF11 is upregulated in TP53-mutated glioma, which drives tumor stemness and chemotherapy resistance while promoting cell cycle progression [68].

The role of the tumor microenvironment (TME) in fostering resistance is increasingly acknowledged. Immune cells, stromal components, and extracellular vesicles within the glioma microenvironment contribute to resistance through various mechanisms. Tumor-associated fibrosis exacerbates glioma chemotherapy resistance by altering cellular communication and increasing stromal stiffness [69]. Tumor-derived exosomes that carry non-coding RNAs, including miRNA, lncRNA, and circRNA, can modulate gene expression in recipient cells, thereby facilitating the development of resistance [70, 71]. For instance, lncRNA LINC00883 enhances the multi-drug resistance phenotype by sponging miR-136, which relieves its inhibitory effect on NEK1 [72]. Additionally, another lncRNA, EPIC1, promotes glioma cell proliferation, migration, and temozolomide (TMZ) resistance by targeting Cdc20 [73].

The abnormal expression of drug efflux pumps, particularly members of the ATP-binding cassette transporter family such as P-glycoprotein (P-gp, ABCB1) and ABCC3, significantly influences the intracellular accumulation of chemotherapy drugs. Notably, ABCC3 is overexpressed in high-grade glioma and correlates with decreased patient survival and resistance to TMZ [74]. Moreover, microRNAs and long non-coding RNAs (lncRNAs) modulate the expression of proteins associated with multi-drug resistance, thereby contributing to efflux pump-mediated resistance mechanisms [75].

**Fig. 3:**
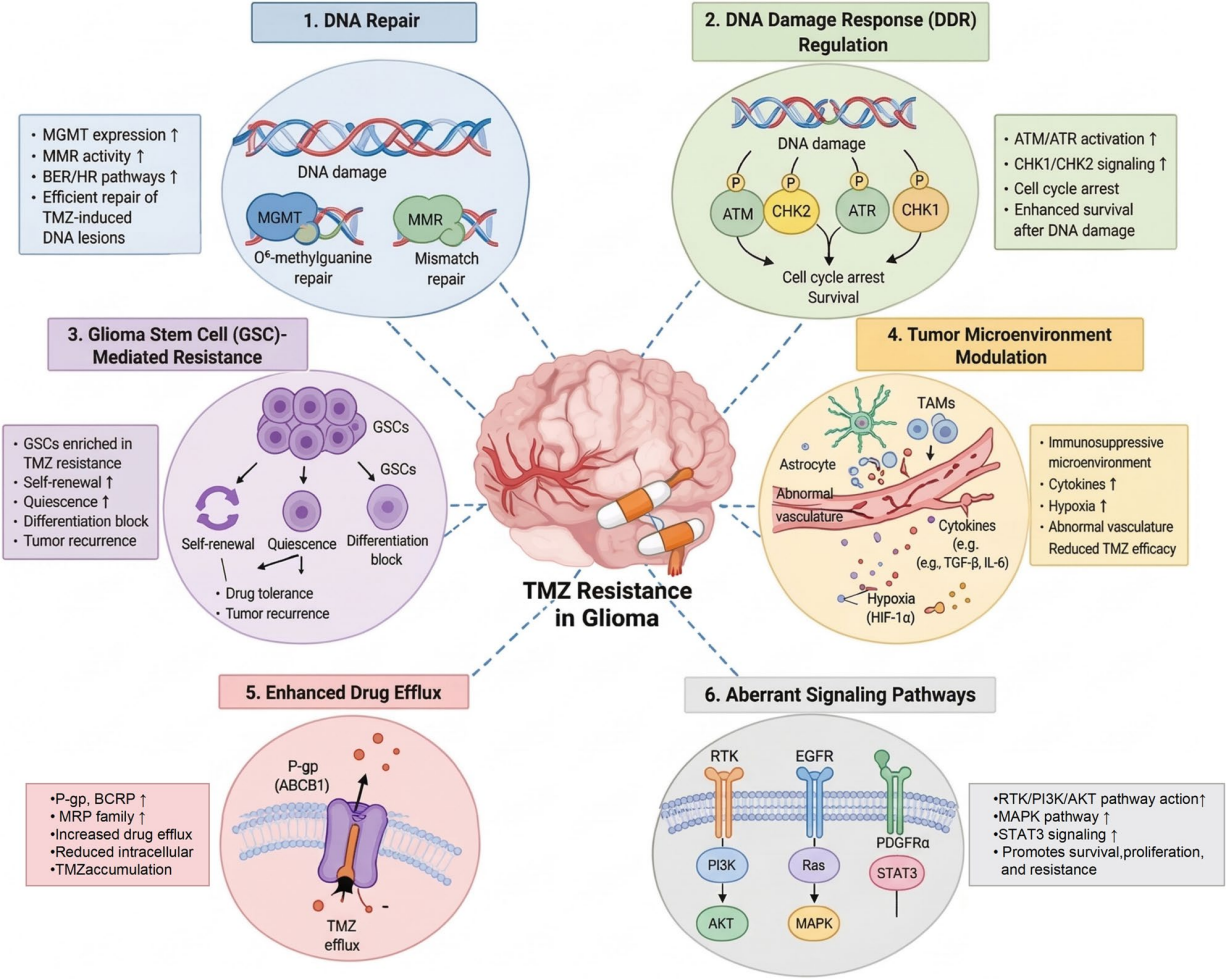
Schematic overview of the major mechanisms underlying TMZresistance in glioma, including DNA repair, DNA damage response regulation, glioma stem cell -mediated resistance, tumor microenvironment modulation, enhanced drug efflux, and aberrant signaling pathways

Abnormal signaling pathways are critical contributors to the development of resistance. Several pathways, including PI3K/AKT, Wnt/β-catenin, NF-κB, and AKT/mTOR, influence tumor cell survival, growth, and treatment resistance [76–78]. For instance, CCL2 enhances glycolysis and TMZresistance by activating the AKT signaling pathway [76]. Additionally, CBX2 stimulates AKT/mTOR signaling through EZH2-mediated silencing of PTEN, thereby promoting proliferation and resistance to TMZ [77]. Furthermore, HAS2 upregulation increases glioma cell proliferation and resistance via the c-myc pathway [79]. Hypoxia-induced upregulation of miR-1290 inhibits PLCB1, which activates Wnt signaling and further contributes to resistance [80, 81]. Fig. 3 illustrates the core mechanisms of TMZresistance in glioma.

### Nanoparticle-mediated drug synergistic delivery

The rapid development of nanoparticle technology offers a versatile platform for co-delivery of chemotherapeutics and resistance-reversal agents, enabling synergistic antitumor effects. Their tunable physicochemical properties (e.g., size, shape, surface charge, and functionalization) facilitate penetration of biological barriers such as the blood–brain barrier and enhance tumor-targeted delivery [82]. Co-encapsulation of agents (e.g., the BRD4 degrader ARV-825) within a single nanoparticle enables synchronized release and maintains stable in vivo drug levels, thereby reducing pharmacokinetic mismatches associated with separate administration [83].

Designing nanoparticle systems for synergistic drug delivery commonly uses lipid, polymeric, or inorganic scaffolds to form stable carriers. Polymeric nanoparticles are particularly advantageous due to their tunable size and surface properties, as well as their ability to enable stimulus-responsive drug release (e.g., pH or enzyme sensitivity) [82, 84]. Surface functionalization with targeting ligands, such as tumor-specific antibodies or peptides, further enhances selective uptake and tumor accumulation [85]. For instance, chitosan-based nanoparticles co-loaded with the BRD4 degrader ARV-825 improve intracellular drug accumulation, overcome resistance, and significantly suppress tumor growth [85].

Nanoparticle systems enhance tumor drug accumulation through multiple mechanisms. They exploit passive targeting (EPR effect) and active targeting (ligand-receptor mediated uptake) [86], protect drugs from in vivo degradation to improve bioavailability and circulation [83], and enable stimulus-responsive release (e.g., pH- or enzyme-triggered) within tumor cells, boosting intracellular drug levels while minimizing toxicity [82, 84]. For example, nanoparticles co-delivering cisplatin and resistance-reversing agents increase tumor drug accumulation, induce apoptosis, and help overcome multidrug resistance [87].

A key advantage of nanoparticle-mediated multi-drug delivery is enhanced synergistic antitumor efficacy. Synergy arises from complementary mechanisms, such as chemotherapeutics inducing DNA damage while resistance-reversal agents inhibit drug-efflux proteins via resistance-related pathways, together boosting cytotoxicity [88]. Nanoparticles also allow spatiotemporally synchronized release, maintaining optimal drug ratios and improving the stability of synergistic effects [89]. Consequently, nanoparticle-based drug combinations demonstrate superior tumor suppression and lower systemic toxicity compared with single agents or free drug mixtures in vitro and in vivo [83, 88].

In summary, nanoparticle-mediated multi-drug synergistic delivery systems enable efficient co-delivery of chemotherapeutics and resistance-reversing agents by precisely controlling particle size, surface functionalization, and drug-loading ratios. These designs overcome limitations of conventional administration, increase intratumoral drug accumulation, and produce synergistic antitumor effects, thereby offering a promising strategy to surmount the glioma blood-brain barrier and drug resistance and providing a solid foundation for future precision cancer therapy [82, 83, 85, 87, 89].

### New strategies for nanoparticle-induced programmed cell death

In recent years, nanoparticles have emerged as a promising strategy to overcome glioma resistance by triggering multiple forms of programmed cell death—pyroptosis, ferroptosis, and apoptosis—thereby bypassing tumor resistance to conventional therapies. Pyroptosis is an inflammation-associated death that stimulates immune responses; ferroptosis involves iron-catalyzed lipid peroxidation; and apoptosis is executed via regulated intracellular signaling. By modulating oxidative stress, nanoparticles can activate these pathways [90, 91]. For example, copper nanoparticles induce programmed cell death in bacteria, suggesting similar mechanisms may operate in tumor cells [90]. Toxicological studies further support that nanoparticles, particularly via ferroptosis, can effectively promote tumor cell death [91]. This multimodal approach offers a potent strategy to enhance glioma treatment efficacy.

Iron- and selenium-based nanoparticles have attracted attention for their ability to modulate oxidative stress and programmed cell death. Silver-coated zero-valent iron nanoparticles (ZVI@Ag) selectively elevate ROS in cancer cells, activating both apoptosis and autophagy while remaining biocompatible with normal cells, highlighting their therapeutic potential [92]. Iron-based nanoparticles can also deplete intracellular glutathione, inducing ferroptosis and enhancing antitumor effects through Beclin1/ATG5-dependent autophagy [93].

Selenium nanoparticles, noted for low toxicity and good biocompatibility, regulate intracellular autophagy and promote tumor cell death. They exert anticancer effects by altering redox balance, antioxidant enzyme activity, and related signaling pathways, and can enhance therapeutic efficacy when used as drug carriers [94]. Together, iron- and selenium-based nanoparticles disrupt glioma resistance mechanisms and promote programmed cell death via oxidative stress and death-signaling modulation, offering promising strategies for glioma therapy.

### Nanocarriers regulating tumor microenvironment to reverse resistance

Nanoparticles are emerging as key carriers in tumor therapy because they offer precise targeting, controllable drug release, and modulation of the tumor microenvironment (TME). Immunosuppression, redox imbalance, and abnormal angiogenesis within the TME drive therapeutic resistance. Functionalized nanocarriers can be engineered to address these specific pathological features, thereby relieving immunosuppression, alleviating hypoxia, and normalizing vasculature to improve drug sensitivity and overall treatment efficacy.

Regulating the tumor immune microenvironment to relieve immunosuppression and enhance immunotherapy is a major research focus. Tumors contain immunosuppressive cells, such as TAMs and regulatory T cells, and express checkpoint molecules that limit therapeutic efficacy. Nanoparticles enable targeted delivery of immune activators, checkpoint inhibitors, or siRNAs to remodel this environment. For example, nanoliposomes co-delivering IDO1 siRNA and immunogenic cell death (ICD) inducers suppress IDO1-mediated immunosuppression, induce ICD, increase CD8⁺ T cell infiltration, and reduce regulatory T cells, thereby improving immunotherapy outcomes [95]. Similarly, nanoparticle-based combinations of chemotherapy, checkpoint blockade, and TGF-β1 silencing can reverse adaptive immunosuppression and enhance antitumor immunity [96]. These studies highlight the potential of nanoparticles to both optimize drug delivery and actively reprogram the tumor immune milieu.

Nanocarriers have strong potential to modulate tumor hypoxia, angiogenesis, and redox homeostasis. Tumor hypoxia promotes multidrug resistance via HIF-1α activation and downstream pathways such as VEGF, driving abnormal neovascularization and tumor progression. Multifunctional nanoparticles can oxygenate tumors, suppress HIF-1α, and inhibit VEGF/EGFR signaling, thereby alleviating hypoxia and enhancing chemosensitivity. For example, oxygen-protein-enriched nanoparticles delivering osimertinib and ginsenoside Rg3 reduce hypoxia and inhibit HIF-1α and VEGF/EGFR pathways, overcoming drug resistance [97]. Similarly, MnO_2_-coated nanoparticles convert H_2_O_2_ to O_2_, relieve hypoxia, and shift macrophages from M2- to M1-like polarization, improving immune modulation and chemotherapy efficacy [98, 99]. Redox-responsive nanoparticles also exploit the tumor microenvironment, releasing drugs selectively in acidic, glutathione-rich conditions to increase intracellular accumulation and counteract multidrug resistance [100–102].

**Fig. 4:**
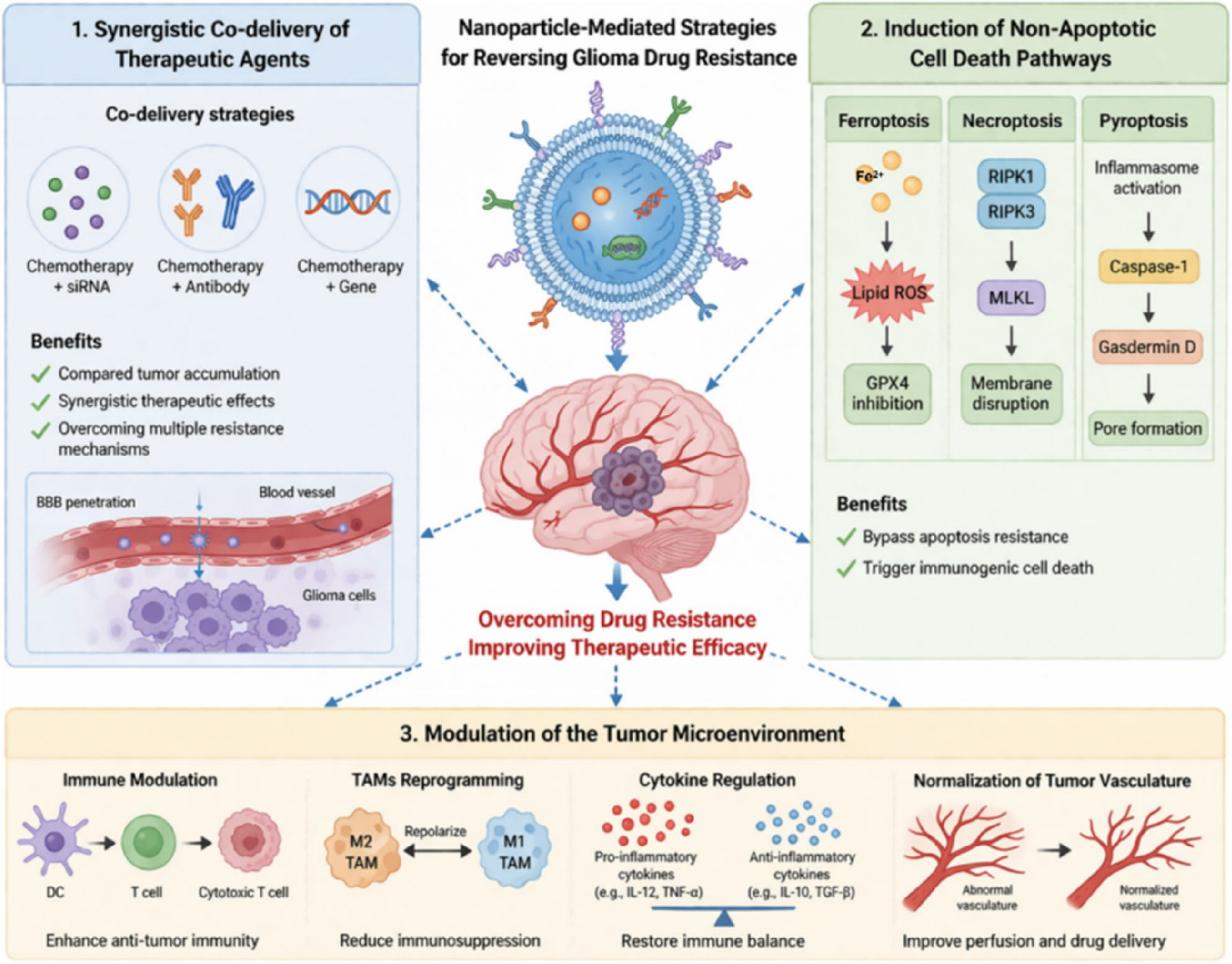
Schematic overview of nanoparticle-mediated strategies for reversing glioma drug resistance, including synergistic co-delivery of therapeutic agents, induction of non-apoptotic cell death pathways, and modulation of the tumor microenvironment

In summary, nanocarrier strategies to overcome tumor resistance focus on modulating the tumor microenvironment through multiple complementary mechanisms: (1) targeting immunosuppressive cells and checkpoints to enhance antitumor immunity and improve immunotherapy outcomes; (2) alleviating hypoxia and inhibiting HIF-1α/VEGF/EGFR signaling to increase chemosensitivity; (3) exploiting redox imbalance to trigger responsive drug release and enhance intracellular accumulation; and (4) normalizing angiogenesis to reduce interstitial pressure, improve drug penetration, and facilitate immune-cell infiltration [103–105]. Figure 4 illustrates these nanoparticle-based strategies for targeting temozolomide resistance in glioma. Figure 4 illustrates the strategic approaches using nanoparticles to target temozolomide resistance in glioma.

While nanoparticle-based strategies have shown promising potential in overcoming specific mechanisms of glioma drug resistance, it is increasingly recognized that targeting a single pathway is often insufficient due to the highly heterogeneous and adaptive nature of glioma. Therefore, recent research has shifted toward the development of multimodal therapeutic strategies that integrate nanoparticle-mediated drug delivery with additional treatment modalities, such as photothermal therapy, photodynamic therapy, and immunotherapy. These combined approaches aim to enhance therapeutic efficacy by simultaneously targeting multiple resistance mechanisms and improving tumor sensitivity to treatment.

## Nanoparticle-assisted multimodal therapeutic strategies

### Photothermal Therapy (PTT) and Photodynamic Therapy (PDT)

Photothermal therapy (PTT) and photodynamic therapy (PDT) are emerging, nanotechnology-assisted approaches for glioma treatment that offer minimal invasiveness, targeting, and controllability. The use of nanoparticles in these phototherapies, especially within the near-infrared (NIR) range and notably the second near-infrared window (NIR-II, 1000–1700 nm), is crucial for enhancing penetration depth and therapeutic efficacy.

Firstly, for photothermal therapy (PTT), the photothermal conversion efficiency of nanoparticles is a key factor influencing therapeutic outcomes. Most conventional photothermal systems operate in the NIR-I window (700–900 nm). For example, semiconductor polymer nanoparticles irradiated with an 808 nm laser exhibit high photothermal conversion efficiency and effective tumor ablation in glioma models [106].

In contrast, recent studies have explored photothermal therapy in the NIR-II window (1000–1700 nm), which offers deeper tissue penetration and reduced light scattering. Nanoparticles with strong absorption in the NIR-II region have demonstrated improved performance in imaging-guided and synergistic phototherapy for glioma treatment [107, 108].

Second, nanoparticles act as carriers for photosensitizers and, in combination with chemotherapy, enhance antitumor efficacy. Nanocarriers loaded with or functionalized by photosensitive molecules (such as chlorin, porphyrins, indocyanine green, etc.) generate substantial ROS under NIR irradiation, thereby inducing apoptosis in tumor cells. For example, lipid nanoparticles encapsulating indocyanine green (ICG) and coated with glioma cell membranes enable homologous targeting of glioma cells and increase intracellular ROS production and photodynamic cytotoxicity [109]. In another study, a functionalized nanoparticle system integrated photothermal and photodynamic modalities and achieved multimodal synergistic therapy by controlling photosensitizer release and excitation, markedly improving therapeutic efficacy [110, 111].

Nanoparticles can also function as multifunctional platforms that enable synergistic chemo-based therapies. For example, nanoparticles coated with cancer cell membranes and loaded with the photosensitizer plus the chemotherapeutic doxorubicin (DOX) exploit homologous targeting to deliver agents precisely to glioma, and upon NIR-laser activation produce concurrent photothermal and chemotherapeutic effects that markedly improve tumor suppression rates [112]. Likewise, copper sulfide (CuS) nanoparticles offer efficient photothermal conversion and release copper ions that synergize with thiourea-based drugs to produce antitumor activity, indicating their potential for photothermal combination chemotherapy [113, 114].

To overcome the BBB in glioma therapy, several studies have developed biomimetic nanoparticles that use neural progenitor cells or macrophages as carriers to transport photothermal nanoprobes across the BBB for deep glioma localization and treatment [115, 116]. Surface modification with brain‑targeting peptides or cell membrane coatings further enhances nanoparticle penetration and tumor targeting, thereby improving the local efficacy of photothermal and photodynamic therapies [117, 118].

In summary, nanoparticles with efficient NIR-II photothermal conversion that also serve as carriers for photosensitizers to promote ROS generation, together with synergistic effects from combined chemotherapy, position PTT and PDT as promising strategies to overcome the blood-brain barrier and drug resistance in glioma. Future advances in material architecture, biological functionalization of nanoparticle carriers, and multimodal treatment combinations are expected to enable more precise and efficient glioma therapies.

### Ultrasound-responsive and mechanical stimulation therapy

Ultrasound-responsive nanoparticles are an emerging therapeutic platform that converts ultrasound mechanical energy into localized biological effects to enable targeted drug release and mechanical stimulation of tumor cells, thereby enhancing therapeutic efficacy. Designing such nanoparticles requires optimizing both ultrasound sensitivity and controllable drug-release behavior. For example, drug-loaded systems based on organic piezoelectric materials release payloads and generate local electrical stimulation when activated by ultrasound, which triggers apoptosis and anti-proliferative signaling and suppresses migration and invasion of drug-resistant glioma cells. These nanoparticles are often functionalized with apolipoprotein E (ApoE) peptides to improve blood-brain barrier crossing and ensure drug delivery to brain tumor sites. Ultrasound-induced mechanical vibration deforms the nanoparticles, triggers drug release, and produces a local electric field that stimulates tumor cells, yielding a synergistic anti-cancer effect from combined chemotherapy and mechanical stimulation [119].

Lipid -polymer composite nanocarriers have also shown excellent drug-loading and ultrasound-triggered release properties. Liposomes composed mainly of oleic acid effectively encapsulate TMZand release the drug via rupture at relatively low acoustic pressures, without inducing significant brain edema or inflammatory responses in vivo. This work supports the use of ultrasound-responsive liposomes for targeted glioma therapy and highlights the potential of ultrasound-induced mechanical stimulation to promote drug release [120].

The use of piezoelectric nanoparticles introduces a novel approach to combined therapy by converting mechanical energy into electrical signals. Under ultrasound activation, these materials generate local microelectric fields that modulate intracellular signaling, promote apoptosis in tumor cells, and suppress their migration. Functionalized piezoelectric nanoparticles paired with ultrasound vibrations enable targeted drug release and provide electrical stimulation that increases tumor-cell sensitivity and immune response, thereby markedly reducing drug resistance and invasiveness [121].

In summary, ultrasound-responsive nanoparticles enable precise drug release and targeted stimulation of tumor cells, thereby improving glioma therapy by overcoming physical barriers and treatment resistance. Future approaches are expected to become more precise and minimally invasive.

### Magnetic-directed nanoparticles and targeted therapy

Magnetic drug carriers (MDCs) use external magnetic fields to steer therapeutic vectors precisely to lesion sites, thereby improving the efficacy and safety of targeted therapy. Magnetic nanoparticles (MNPs) form the functional core of these guidance systems; under applied magnetic fields they overcome physiological barriers and blood-flow shear forces, enabling efficient accumulation within tumor regions. This localized accumulation promotes targeted drug release and reduces toxic effects on healthy tissues. Recent research on magnetic guidance for tumor therapy has advanced significantly, with notable promise for treating brain tumors such as glioma.

Design of magnetic nanoparticles must balance size, stability, and magnetic responsiveness. Chitosan-coated magnetic nanoparticles with diameters of about 10–20 nanometers show favorable stability and controllability, withstand shear stress in blood flow, and tolerate varying magnetic-field strengths in in vitro chip models that simulate tumor microenvironments, thereby enabling efficient magnetic targeting [122]. These microfluidic models embed tumor and endothelial cells in a hydrogel matrix to recapitulate vessel -tumor interactions and provide a reliable platform for preclinical screening of magnetic guidance therapies. Embedding magnetic nanoparticles within natural bacteria produces self-propelled “nano-bacterial magnets” that actively penetrate tumor hypoxic cores, perform effective magnetic hyperthermia, and markedly increase pancreatic cancer cell mortality, producing substantial tumor suppression in in vivo models [123].

Iron oxide nanoparticles (IONPs) are central to magnetic-guided drug delivery and cancer therapy because they combine biocompatibility, magnetic responsiveness, and imaging capability. They function as drug carriers that can be precisely localized with an external magnetic field and can also promote ferroptosis via ROS generation, thereby enhancing antitumor efficacy [124, 125]. In non-small cell lung cancer, researchers synthesized functionalized magnetic nanoparticles conjugated to anticancer fatty acids and other ligands to target tumor angiogenesis markers, improving therapeutic potency and tissue selectivity [126]. PEGylated superparamagnetic iron oxide nanoparticle delivery systems paired with photothermal therapy demonstrated strong cellular uptake and cytotoxicity in breast cancer cells, suggesting promise as multimodal treatment platforms [127].

A key advantage of magnetically directed nanoparticles is their compatibility with magnetic resonance imaging (MRI), which enables real-time monitoring and guidance during therapy. The magnetic responsiveness of these nanoparticles enhances MRI contrast in tumor tissues, aiding precise localization and evaluation of drug distribution. For example, iron oxide nanoparticles functionalized with targeting ligands both increase tumor accumulation and permit MRI-based tumor imaging, which facilitates individualized adjustment of treatment plans [128, 129]. In glioma therapy, functionalized magnetic nanoparticles exploit glucose transporter (GLUT)-mediated endocytosis together with induced mild hypoglycemia to penetrate and target tumor microvessels efficiently; this approach markedly delays tumor growth and supports the feasibility and efficacy of MRI-guided magnetic hyperthermia [130].

Magnetically guided drug-release systems continue to be refined. For example, magnetic nanoparticles coated with multifunctional organic layers enable responsive drug release in acidic and reductive tumor microenvironments, thereby improving treatment specificity and efficacy [131]. Composite nanoparticle platforms that combine the photothermal and magnetic heating effects of magnetic nanoparticles enable multimodal tumor therapy and further enhance therapeutic outcomes [132].

**Fig. 5:**
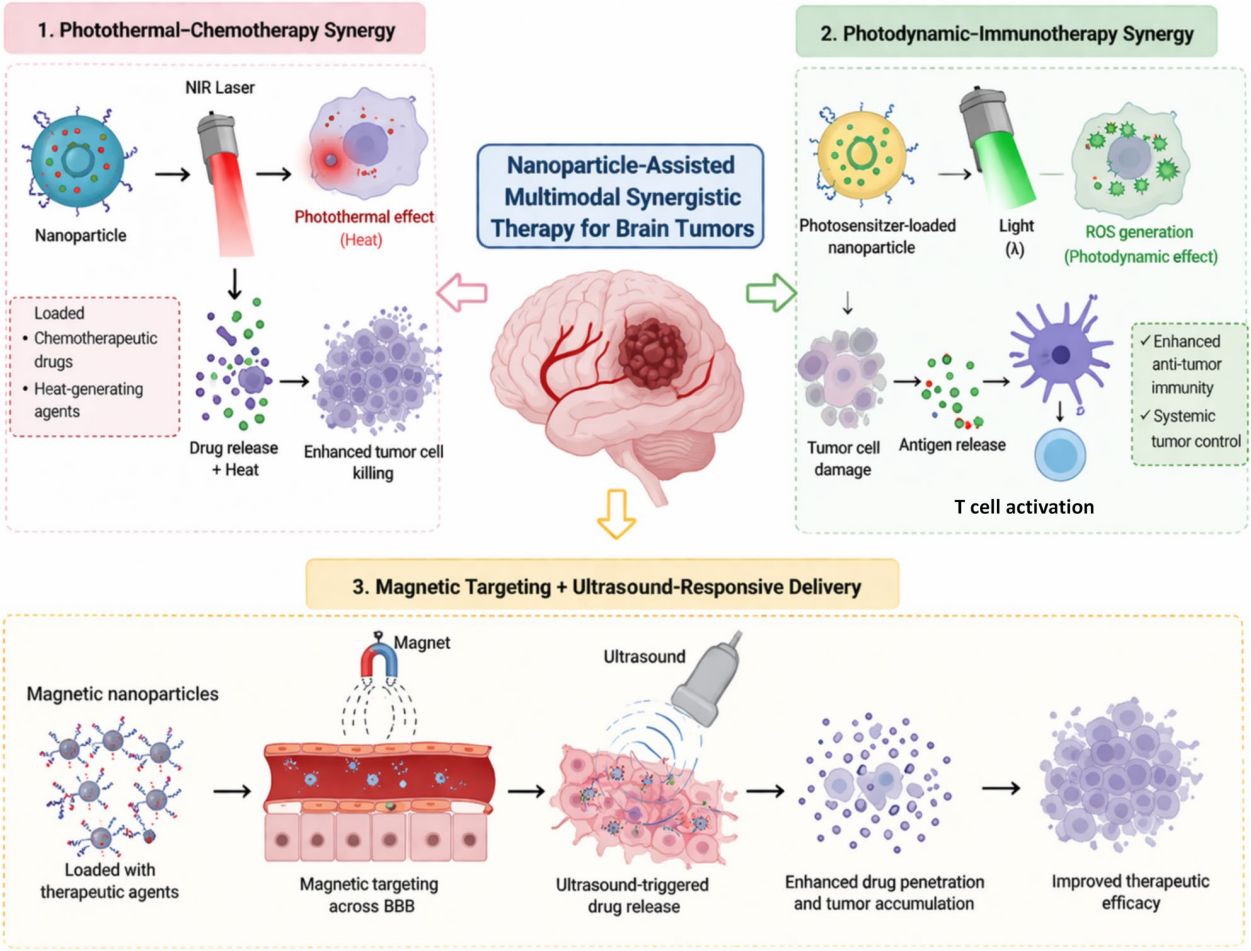
Schematic overview of nanoparticle-assisted multimodal synergistic therapeutic strategies for brain tumors, including photothermal-chemotherapy synergy, photodynamic-immunotherapy synergy, and magnetic targeting combined with ultrasound-responsive delivery

In summary, magnetically guided nanoparticles enable targeted drug delivery under external magnetic fields, effectively addressing the blood-brain barrier and therapeutic resistance in glioma. When combined with MRI, these nanoparticles permit real-time monitoring and adjustment of treatment, thereby advancing precision medicine. A schematic of the nanoparticle-assisted multimodal synergistic treatment strategy for brain tumors is shown in Fig. 5.

### Preclinical research progress of multifunctional nanoplatforms

Multifunctional nanoplatforms have advanced glioma research; by integrating modalities such as chemotherapy and immunotherapy to increase efficacy and overcome physiological barriers. Studies demonstrate that these platforms can traverse the BBB, selectively target tumor tissue, and improve survival when used in combination regimens.

For example, PLGA-PEG nanoparticles encapsulating doxorubicin (DOX) and the near-infrared dye IR780 produced synergy between chemotherapy and photothermal therapy in a mouse model of triple-negative breast cancer, induced immunogenic cell death (ICD) and, when combined with anti-PD-1 immune checkpoint blockade, markedly activated systemic antitumor immune responses [133]. Similar approaches have been applied to glioma where multifunctional polydopamine nanoplatforms loaded with DOX and ICG; enabled efficient targeted delivery and multi-stimuli responsive drug release via targeting peptide modifications, substantially enhancing therapeutic efficacy and reducing tumor cell survival rates [134].

Gold nanorods (AuNRs) cloaked with red blood cell membranes and decorated with antibodies functioned as a biomimetic nanoprobe that combined gene interference with photothermal therapy, yielding notable antitumor activity against pancreatic cancer and offering guidance for designing multifunctional biomimetic nanoplatforms [135]. In immunotherapy, a multidimensional nanoplatform that integrates ferroptosis inducers with immunomodulatory agents enhanced immune cell infiltration and restored immune surveillance by reshaping the tumor microenvironment, thereby addressing the limited immune activation of standalone ferroptosis treatments and demonstrating marked therapeutic potential [136]. Collectively, these studies indicate that such nanoplatforms are effective and well tolerated in animal models, showing good biocompatibility and minimal systemic toxicity.

The development of biomimetic nanocarriers has produced new breakthroughs in glioma therapy. Nanoplatforms formed by fusing various cell membranes (such as red blood cell membranes, tumor cell membranes, and microglial cell membranes) both prolong circulation and reduce immune clearance, while enabling active glioma targeting and blood-brain barrier penetration, thereby increasing drug accumulation and release at tumor sites [137–139]. These platforms frequently incorporate multiple stimulus-responsive mechanisms-pH, light, and enzyme triggers-to enable controlled drug release and thus minimize damage to normal tissues.

In designing multimodal nanoplatforms, researchers currently emphasize four optimization goals: improving blood-brain barrier penetration efficiency, enhancing tumor targeting, achieving multifunctional synergistic therapies, and ensuring biosafety. Specific strategies include:

(1) Biomimetic and surface modification: Coating nanoparticles with membranes from red blood cells, tumor cells, or immune cells confers immune evasion and target-recognition properties, thereby prolonging circulation time and increasing brain accumulation [137, 139, 140]. Combining these strategies with active targeting ligands, such as peptides or antibodies against CD44, EGFR, or PDGFRβ, further enhances nanoparticle recognition and uptake by tumor cells and tumor-associated fibroblasts [112, 141].
(2) Multifunctional integration: Combine chemotherapy, photothermal therapy, photodynamic therapy, gene therapy, and immunomodulation into multimodal regimens to produce synergistic tumor cell killing and to overcome drug resistance and tumor heterogeneity associated with single-modality treatments [133, 136, 142]. Design intelligent nanocarriers that respond to tumor microenvironment cues (such as pH, GSH, ROS, and enzymes) to enable targeted, controlled drug release and thereby reduce systemic toxicity [143, 144].
(3) Imaging -guided nanoplatforms that integrate drug delivery with MRI, PA, NIR fluorescence, and other modalities enable real-time monitoring and precise localization during treatment, thereby improving controllability and safety [138, 139, 145].
(4) Biocompatibility and metabolism: Prioritize tbiodegradable materials or natural polymers (for example,,polysaccharides and proteins) when constructing nanoplatforms to minimize the risk of long-term accumulation in the body and to ensure favorable safety and metabolic profiles [137, 146, 147].
(5) Artificial intelligence-assisted design: Employ computational simulations and artificial intelligence to optimize material selection, structural design, and functional integration of nanoparticles, thereby enhancing carrier penetration, targeting accuracy, and therapeutic efficacy [136].

In summary, preclinical research on multifunctional nanoplatforms indicates that rational design and the integration of complementary therapeutic modalities, together with precise targeting and responsiveness to the tumor microenvironment, have substantially improved treatment outcomes for malignant tumors such as glioma. Future efforts will prioritize biosafety, deeper multifunctional integration, and intelligent design to accelerate clinical translation of these nanoplatforms.

## Challenges and future directions of nanoparticles in clinical translation

### Biosafety and toxicological assessment

Nanoparticles are important carriers and therapeutic agents for glioma treatment, and their biosafety and toxicological profiles are critical for clinical translation. In vivo metabolism, biodistribution, and toxicity are complex and depend on factors such as material, size, shape, surface modification, and administration route. Studies have extensively examined a range of nanoparticles-including silver nanoparticles (AgNPs), mesoporous silica nanoparticles (MSNs), iron oxide nanoparticles (Fe3O4NPs), gold nanoparticles (AuNPs), polymer nanoparticles, and Prussian blue nanoparticles (PB NPs)-which show substantial differences in biocompatibility and toxicological behavior.

Silver nanoparticles have attracted broad attention for their distinctive physicochemical properties and medical uses, including antibacterial and anticancer applications, but their potential toxicity to cells, tissues, and organs remains a concern. Toxic effects-principally driven by silver ion release, ROS generation, and disruption of membrane integrity-depend strongly on nanoparticle morphology and surface modification. For example, protective surface coatings such as lipoic acid (AgNPsLA) markedly reduce toxicity in the vascular microenvironment, lower ROS production, and decrease apoptosis, thereby improving safety [148, 149]. Conversely, silver nanoparticles can trigger endoplasmic reticulum stress responses in the brain tissue of juvenile animals, indicating that their neurotoxic potential warrants careful scrutiny [150].

Mesoporous silica nanoparticles (MSNs) are central to nanoparticle drug delivery because they offer high drug-loading capacity and controlled release. Their biosafety depends strongly on physicochemical attributes such as particle size, shape, surface charge, and surface modifications. In vivo studies show that MSNs mainly accumulate in the lungs, liver, and spleen, and high-dose administration can cause acute toxicity that is largely attributable to physical effects and is associated with low immunotoxicity [151, 152]. Surface PEGylation enhances the biocompatibility and degradability of MSNs, which may facilitate their clinical translation.

Iron oxide nanoparticles (Fe_3_O_4_NPs) exhibit strong magnetic responsiveness and catalytic activity, which makes them attractive for tumor diagnosis and therapy. Green biosynthesis of Fe_3_O_4_NPs enhances stability and functionality while lowering environmental impact, but their peroxidase-like catalytic activity can generate reactive oxygen species (ROS) and raise potential biological toxicity [150, 153]. Consequently, standardized toxicological assessments and safety guidelines should be established promptly.

AuNPs display prolonged blood circulation in vivo and primarily accumulate in the liver and spleen; they can induce apoptosis and short-term metabolic disturbances, while their long-term toxicity is relatively low [154]. Surface modification with polyethylene glycol (PEG) further improves biocompatibility and reduces DNA damage and inflammatory responses, indicating favorable safety profiles.

Polymer nanoparticles, including poly (β-amino ester) (PBAE) and polymer-lipid hybrid formulations. exhibit good cellular tolerance and in vivo safety. By binding antioxidant molecules such as glutathione (GSH), they can effectively reduce intracellular ROS, alleviate cytotoxicity, and enhance gene delivery efficiency [155].

Prussian blue nanoparticles (PB NPs) display morphology- and charge-dependent effects on biocompatibility: larger size and increased positive charge reduce cell viability and worsen liver and kidney function, yet they do not produce significant organ retention in vivo and are rapidly metabolized [156]. Other particles, such as selenium nanoparticles (SeNPs) synthesized by green methods, show favorable biosafety and confer radiation protection [157, 158].

Long-term safety and immunogenicity of nanoparticles are critical for clinical translation. By forming a protein corona, nanoparticles can modulate immune activity and thereby provoke immune stimulation or suppression, apoptosis, and inflammatory responses [159, 160]. At high doses or with prolonged exposure, some nanoparticles produce liver and kidney toxicity, neurotoxicity, and reproductive-system damage [161–163]. Consequently, nanoparticle drug development must include a systematic toxicological assessment encompassing acute and chronic toxicity, immunotoxicity, genotoxicity, and pharmacokinetics.

Biosafety and toxicological assessments of nanoparticles must integrate material properties with in vivo behavior, covering studies from in vitro assays to animal models. Optimizing surface modifications and controlling particle size improve safety and thereby strengthen nanoparticle-based strategies for glioma. Successful clinical translation of nanoparticles as anti-glioma agents will depend on unified evaluation standards and long-term safety studies. Table 1.

**Table 1:** Comparison of biosafety and toxicological profiles of nanoparticles for glioma therapy

Type of Nanoparticles	Core Characteristics	Main Toxicity Mechanisms/Potential Risks	Safety Optimization Strategies
AgNPs	Unique physicochemical properties; antibacterial and anticancer activities	Silver ion release, ROS generation, cell membrane damage; endoplasmic reticulum stress in juvenile brain tissue (neurotoxicity)	Lipoic acid modification (AgNPsLA) to reduce vascular toxicity, ROS production and cell apoptosis
MSNs	Excellent drug loading capacity and controlled release performance	High-dose administration induces acute toxicity (related to physical effects); low immunotoxicity	Surface PEGylation modification
Fe₃O₄NPs	Excellent magnetic responsiveness and catalytic activity; green biosynthesis reduces environmental impact	Catalytic peroxidase-like activity induces ROS generation	Establish standardized toxicological evaluation and safety criteria
AuNPs	Long blood circulation time in vivo	Short-term exposure causes cell apoptosis and metabolic disorders; relatively low long-term toxicity	PEG modification to enhance biocompatibility and reduce DNA damage and inflammatory responses
Polymeric Nanoparticles	Good cellular tolerance and in vivo safety; favorable for gene delivery	Potential ROS-associated cytotoxicity	Combination with glutathione (GSH) to reduce intracellular ROS levels and alleviate cytotoxicity
PB NPs	Rapid metabolic rate in vivo	Increased particle size and positive charge reduce cell viability and exacerbate hepatorenal damage	Regulate particle size and surface charge

### Preparation processes and scalable production

The scalable preparation of nanoparticles is essential for clinical and industrial applications, with an emphasis on batch consistency, quality control, cost-effectiveness, and resolving industrial bottlenecks. Contemporary approaches encompass microfluidics, emulsions, solvent evaporation, co-precipitation, solid-phase synthesis, gas-phase synthesis, and green biosynthesis, and these methods continue to be optimized for diverse materials and uses.

First, consistent quality control of nanoparticle batch preparation is essential for scalable production. For PLGA nanoparticles, drug encapsulation efficiency and loading capacity are key attributes. Machine learning combined with microfluidic tuning can optimize these attributes using a dataset of over 300 formulations, enabling precise control and consistent quality [164]. Microfluidic methods are also widely used to produce polymer and lipid nanoparticles because they offer tight process control and scalability [165, 166]. For example, continuous-flow LNP manufacturing yields uniform particle sizes and high encapsulation efficiencies, allowing bulk production that meets clinical dosing requirements [167].

Second, scalable production faces challenges of cost, equipment requirements, process complexity, and environmental impact; conventional approaches often rely on solvents and generate pollution. Green synthesis routes, such as biosynthesis and plant-extract methods, provide eco-friendly and cost-effective alternatives for nanoparticle manufacture [168, 169]. Emerging platforms-including continuous‑flow reactors and microfluidic chips-improve efficiency and consistency while reducing energy consumption and waste. For example, microfluidic systems enable precise control over silk fibroin nanoparticle formation and support GMP-level production [170]. Concurrently, high-pressure homogenization, static mixers, and ultrasonic emulsification have been implemented for industrial-scale fabrication of solid lipid nanoparticles (SLNs), addressing scalability and maintaining quality stability for drug delivery applications [171, 172].

Despite progress, significant challenges remain for scalable nanoparticle production. First, nanoparticle physicochemical properties change with scale-dependent parameters, which can alter performance and safety. For example, silver nanoparticles synthesized by ultrasonic-assisted methods require specific conditions to maximize yield when free radical scavenger concentrations vary [173]. Second, practical obstacles such as line blockages, poor dispersion stability, solvent residues, purification difficulties, and storage constraints impede large-scale manufacturing [174, 175]. Finally, equipment selection and strict standardization of process parameters are critical for batch-to-batch consistency, as exemplified by stable polymer nanoparticle production using co-axial jet mixers and uniform lipid nanoparticle synthesis via microfluidic technology [176].

Finally, economic viability and sustainability are essential for scalable production, and life cycle assessment can guide synthesis pathway optimization to lower costs and environmental impact [177]. For example, aerosol-based synthesis of carbon nanoparticles offers efficiency and reduced ecological burden. Industrial-scale production must also address raw material supply chains, capital investment in equipment, safety protocols, and regulatory compliance to meet standards.

In summary, nanoparticle preparation and production must balance feasibility, cost, and environmental impact while maintaining product quality. Future progress in intelligent process control, green synthesis methods, continuous-flow reactors, and multiscale design will facilitate their industrial scale-up and clinical translation.

### Clinical trial design and regulatory oversight

Current status of clinical trials of nanomedicines in glioma treatment:

Significant progress has been achieved in nanomedicine trials for glioma, where biocompatible and stable nanoparticles such as lipid, polymer, gold, and biomimetic types have been developed to overcome the BBB and BTB barriers [178, 179]. However, the actual delivery efficiency across the BBB in clinical settings remains limited, and heterogeneous barrier integrity within glioma may lead to uneven nanoparticle distribution.

Current clinical nanomedicine programs emphasize safety and preliminary efficacy and typically use dose-escalation Phase I designs to determine the maximum tolerated dose. For example, lipid nanoparticle formulations have been administered intranasally to enhance brain drug concentrations [180]. In parallel, trials are combining nanoparticles with focused ultrasound to transiently open the blood-brain barrier and enable spatially precise drug delivery [181]. Gold nanoparticle-based approaches paired with radiotherapy or photothermal therapy are in early clinical testing and have demonstrated safety and signals of efficacy [182]. Nevertheless, most of these studies remain at early clinical stages, and robust evidence of therapeutic benefit in large patient cohorts is still lacking.

Clinical development nonetheless faces challenges, including incomplete characterization of nanoparticle behavior, patient heterogeneity, and concerns about long-term safety; accordingly, efforts now prioritize patient selection, targeting optimization, and integration of advanced imaging [183, 184]. In addition, discrepancies between preclinical and clinical outcomes are frequently observed, as promising results in vitro or in small animal models are not always reproducible in human patients due to the complexity of the tumor microenvironment and physiological barriers.

Regulatory approval pathways for nanomedicines are more complex than those for conventional drugs because of unique physicochemical properties and safety assessment needs, and agencies such as the FDA and EMA stress systematic evaluation of these factors [185]. Regulatory approval requires detailed characterization of nanoparticles, including size distribution, surface properties, drug loading and release, stability, and batch-to-batch consistency. In vivo studies of absorption, distribution, metabolism, and excretion (ADME), together with assessments of immune responses, organ toxicity, and long-term accumulation risks, are also required [184].

Preclinical evaluation of nanomedicines combines in vitro and animal models with advanced imaging to verify safety and tumor-targeting efficacy. However, commonly invoked mechanisms such as the enhanced permeability and retention (EPR) effect remain controversial in clinical contexts, particularly in brain tumors, where the BBB is only partially disrupted and highly heterogeneous. This limitation further contributes to the gap between preclinical success and clinical translation.

Clinical trials demand enhanced monitoring of pharmacokinetics and immunogenicity to ensure patient safety [186]. Overall, despite encouraging progress, the clinical translation of nanomedicines for glioma remains constrained by delivery inefficiency, biological complexity, and regulatory challenges. Future trial design should integrate more clinically relevant models, standardized evaluation criteria, and strategies to improve BBB penetration and targeting specificity, thereby facilitating the successful translation of nanomedicine-based therapies.

### Future research directions and innovative strategies

Future glioma research will emphasize smart nanoparticles and interdisciplinary approaches to enable clinical translation, with a focus on precise drug release and personalized therapies that enhance drug penetration and targeting.

Smart nanoparticles can precisely recognize the glioma microenvironment and release drugs by means of surface functionalization and multi-stimulus responsiveness. For example, nanoparticles that respond to pH shifts, enzymatic activity, or reductive conditions in the tumor milieu trigger on-site drug release, thereby reducing off-target effects in normal brain tissue and improving treatment selectivity and safety [187]. However, the heterogeneity and dynamic nature of the tumor microenvironment may limit the reliability and reproducibility of such stimulus-responsive systems in clinical settings.

In addition, biomimetic nanoparticles cloaked with biological membranes—such as mesenchymal stem cell membranes that confer tumor-homing—prolong circulation time and markedly enhance BBB penetration and tumor accumulation [20, 21]. This biomimetic approach addresses the cell-source limitations and heterogeneity of conventional nanoparticles and facilitates precise postoperative adjuvant therapy. Nevertheless, issues related to large-scale production, membrane source standardization, and potential immunogenicity remain to be fully resolved.

Personalized treatment depends on nanoparticle carriers that respond to tumor heterogeneity and patient-specific differences. Surface modification with targeting ligands, for example transferrin or Angiopep-2 peptide, enables active targeting of glioma cells and improves cellular uptake and therapeutic efficacy [188, 189]. However, variability in receptor expression among patients may affect targeting efficiency and limit universal applicability.

Multifunctional nanoparticle platforms that combine photothermal therapy, photodynamic therapy, gene therapy, and immunotherapy produce synergistic effects and can overcome drug resistance and tumor recurrence [190, 191]. For instance, multi-component nanoparticle-based combination strategies that induce both apoptosis and ferroptosis synergistically eradicate tumor cells and substantially delay disease progression [192]. Despite these promising results, the increased complexity of multifunctional systems may pose challenges for regulatory approval, reproducibility, and clinical translation.

The development of nanomedicine brings together materials science, biology, medicine, and engineering, employing microfluidic chips and 3D blood-brain barrier models to evaluate nanoparticle performance and facilitate clinical translation of therapies [193]. The use of artificial intelligence, big data, and high-throughput screening is expected to enable intelligent, personalized nanoparticle design, optimizing physicochemical properties and biocompatibility while minimizing off-target effects [194, 195]. However, the predictive accuracy of these models and their consistency with in vivo human responses remain areas requiring further validation.

Clinical deployment of nanoparticles must also address biosafety, immunogenicity, in vivo metabolism, and clearance. Biomimetic carriers and nanoparticles coated with natural components exhibit favorable biocompatibility and low immunogenicity, and they are therefore key focuses for future research [20, 21]. Combining nanomedicine with conventional therapies can improve drug efficacy in glioma patients [194, 196].

Overall, while these innovative strategies offer significant promise, their successful clinical translation will depend on overcoming challenges related to scalability, safety, regulatory approval, and inter-patient variability.

The future of glioma nanomedicine lies in smart, personalized nanoparticles whose interdisciplinary design overcomes blood-brain barrier limitations and drug resistance, ultimately improving patient outcomes.

## Conclusion

Nanoparticle technology offers substantial promise for glioma treatment, particularly in traversing the blood-brain barrier and mitigating drug resistance. Advances in material design and functional modification now enable precise targeting and controlled drug release, which enhance therapeutic efficacy while limiting injury to healthy brain tissue.

However, heterogeneity in nanomaterial classes, surface modifications, and drug-loading methods produces inconsistent outcomes and safety profiles across studies. Establishing standardized evaluation criteria-especially for biosafety, long-term toxicity, and immune response-is therefore essential to translate preclinical findings into reliable clinical applications.

Additional challenges include scaling production and satisfying regulatory requirements. Future work should prioritize streamlining synthesis, ensuring batch-to-batch consistency, and performing rigorous clinical trials. Combining nanotechnology with immunology, molecular biology, and gene editing could yield smarter, more personalized therapeutic systems.

In summary, although nanoparticle-based glioma therapy is highly promising, coordinated interdisciplinary efforts and standardized development pathways will be critical to surmount current barriers and achieve safe, effective clinical translation. 

## Data Availability

The data generated and/or analyzed during this study are available from the corresponding author on reasonable request.
